# Disseminating the research findings from the adolescents and adults living with Perinatal HIV (AALPHI) study: an approach from young people living with HIV

**DOI:** 10.1186/s40900-024-00541-x

**Published:** 2024-01-18

**Authors:** Kate Sturgeon, Ali Judd, Tom Burke, Caroline Foster, Diana M. Gibb, Marthe Le Prevost, Warren Mhizha, Conor D. Tweed

**Affiliations:** 1 MRC Clinical Trials Unit at University College London, London, UK; 2 Amplify Consultancy, London, UK; 3 Imperial College Healthcare NHS Trust, London, UK; 4 Youth Trials Board, London, UK; 5 Children’s HIV Association (CHIVA), Bristol, UK

**Keywords:** HIV, Young people, Patient and public involvement, Co-production, Dissemination of research

## Abstract

**Background:**

The Adolescents and Adults Living with Perinatal HIV (AALPHI) study is one of only three cohort studies worldwide evaluating the impact of HIV on young people living with perinatal HIV (PLHIV) relative to a comparable group of HIV negative young people in close relationship with an HIV positive individual, for example, their mother, sibling or partner. This project aimed to engage young people with the AALPHI study findings, help them take ownership, and decide how they would disseminate the key messages to both study participants and to the wider community.

**Methods:**

In brief, 318 PLHIV and 100 HIV negative adolescents participated in AALPHI, where they each were interviewed twice, around two years apart. They were asked a wide range of psychosocial and risk behaviour questions and their cognitive function was assessed. We invited three AALPHI participants and seven members of the Youth Trials Board at the Children’s HIV Association (CHIVA) to attend up to four workshops. They were provided with the key AALPHI research findings and asked to develop them into a format that was accessible and understandable for young people. Some who had not participated before formed a group in the fourth dissemination workshop that confirmed the most important concepts and results.

**Results:**

The young people decided to develop a film and a leaflet about the AALPHI findings and co-produced them with a film maker and graphic designer. Challenges included working with the film maker and the venue for the first three dissemination workshops was an office space which was not ideal.

**Conclusion:**

Engaging young people in the dissemination of the AALPHI findings ensured the results were communicated in a way that was more likely to be relevant, accessible and useful to those affected by the study. This project demonstrates how young people in potentially stigmatised areas of care, such as HIV, can be involved in research dissemination.

## Abstract (plain language summary)

Informing young people of the results of a study in which they participated, in a manner they can understand, is an ethical minimum. Increasingly, young people themselves may be involved in this dissemination activity, to ensure that study results are communicated in a way which is more likely to be relevant, accessible and useful to those directly affected by the study. The Adolescents and Adults Living with Perinatal HIV (AALPHI) study is a cohort study evaluating the impact of HIV on young people living with perinatal HIV (PLHIV) relative to HIV negative young people affected by HIV. This project aimed to engage PLHIV with the AALPHI findings, and help them take ownership of their dissemination, deciding how to communicate key messages to study participants and the wider community. We invited three AALPHI participants and seven members of a Youth Trials Board at the Children’s HIV Association, (CHIVA), to attend four workshops. We provided them with key AALPHI findings and asked them to develop them into an understandable format for young people. They co-produced the content for a film and a leaflet about the results, working with a film maker and graphic designer. The 4th comprised of three workshop participants and seven new participants from CHIVA. This work shows that young PLHIV can be part of the process of evaluating study results and guiding dissemination by creating outputs that align with young people’s priorities. This area of work could be further developed in the future through direct evaluation of participant involvement.

## Background

It is good practice for young people to be informed of the results of the study in which they participate and to learn from the study findings, in a youth-friendly manner. Researchers are increasingly encouraged to involve young people in dissemination activity themselves so that study results include information and are formatted in a way that is more likely to be relevant, accessible and useful to those affected by the study results [1].

The Adolescents and Adults Living with Perinatal HIV (AALPHI) cohort was one of only three cohort studies worldwide (the other two being in the USA and South Africa) evaluating the impact of HIV on young people living with perinatal HIV (PLHIV) relative to HIV negative young people affected by HIV, in England [2, 3]. In total, 318 PLHIV were recruited for the study with a comparison group of 102 HIV negative adolescents who were either siblings of the PLHIV group or who had a parent or partner living with HIV. The PHIV participants were aged 13–21 years and the HIV negative participants were aged 13–23 years and were interviewed twice between 2013 and 2017. Interviews explored a broad range of psychosocial domains and risk behaviours, and cognitive function was assessed. The PLHIV group were very similar to the HIV negative group in terms of cognitive performance, levels of anxiety and depression and self-esteem, giving reassurance to PLHIV about their long-term cognitive and mental health [4, 5].

Many benefits to young people of being involved in research have been cited in the literature, for example the experience may be life enhancing and help increase confidence, self-esteem and the belief that their views matter and lead to change [6, 7]. If PLHIV understand that the findings from AALPHI were broadly positive, they may have reduced anxiety and improved wellbeing as there is something to live for and aspire towards.

Patient involvement also benefits researchers. It improves the trustworthiness of the research for other patients and increases the transparency and relevance of the research [8]. Co-production of participant-facing materials has also been shown to improve recruitment into a study [9] and creative practices can play a role in supporting diverse people to engage in co-production [10].

Patient and Public Involvement (PPI) has been a priority within AALPHI since it began in 2012. Through our established links with the Children’s HIV Association (CHIVA), a group of five PLHIV were gathered to design the study logo, as well as posters advertising the study. We also worked with young people to pilot the interview questions, and gain their feedback on the study methods and interview duration, consent forms, and study materials. They also developed a video explaining the study to potential participants, [11].

In continued recognition of the importance of engaging with study participants, we designed workshops to engage PLHIV and study participants in the design and dissemination of the AALPHI study findings. We hypothesised that this engagement would lead to them feeling a sense of empowerment about their health and increase their confidence to self-advocate to health care professionals.

The PPI work in this project aims to familiarise young people with the AALPHI study findings, help them take ownership and decide how they would disseminate the key messages to PLHIV and HIV negative young people that participated in the study and the wider community.

## Methods

Seven participants were recruited through the Youth Trials Board UK (YTB UK) and CHIVA networks and three young people who had been in AALPHI were invited to attend workshops to develop dissemination materials. They were all young PLHIV and on antiretroviral treatment. The facilitators for all the workshops were affiliated with AALPHI and CHIVA, and had experience in logistics, safeguarding and pastoral care. They facilitated every group discussion in each workshop.

The first three dissemination workshops were held at the MRC Clinical Trials Unit at UCL, in central London, UK and the fourth in Bristol, UK. The second and third workshops were two-day residentials, which meant that young people also had the opportunity to have fun activities and social time with one another. Each session began with introductions and exploration and agreement on ways of working and concluded with a reflection and evaluation exercise which included feedback from participants about how the session had gone. The first three workshops involved the same 10 participants, and they had the task of producing a design for disseminating the findings. Only three of these participated in the final workshop to confirm the most important concepts and results.

### Dissemination workshop 1

In June 2018, the first dissemination workshop was held to set the scene for participants. In this first workshop, the AALPHI findings were explained, and the most appropriate methods to communicate the findings more broadly were discussed and agreed.

The workshop began with a presentation of the main published findings from AALPHI, on: (1) medication and adherence; (2) mental health (depression and anxiety) and ‘how your brain’s doing’ (cognition); (3) sexual behaviour; and (4) transition -moving from paediatric to adult care and the key messages on each. For each topic the participants identified and wrote key messages in a way that they found understandable. Participants were asked to complete a multiple choice questionnaire on what they thought the AALPHI results would show before the key findings were presented. They were then asked to complete the same questionnaire after the presentation to assess if their level of knowledge had increased. The workshop participants were keen to develop a leaflet and short film about the study results, therefore a graphic designer and film maker were sought.

### Dissemination workshop 2

In November 2018, a second dissemination workshop was run over a residential weekend to design the dissemination materials. The first day started with a refresher briefing on the background to the study and previous decisions on dissemination methods. In this second workshop young PLHIV were asked to prioritise the information to be included in the dissemination materials and decide the content and layout of the materials. Young PLHIV were split into working groups and facilitators aimed to build consensus on content for the dissemination materials using group discussion and a mood board, [12]. The young people completed evaluation forms on how they felt the workshop had gone. The agreed points were fed back to the graphic designer and film maker via a call with the facilitators and photos of the mood boards were also sent to them.

### Dissemination workshop 3

A third dissemination workshop was held in February 2019 over a residential weekend with the young PLHIV to complete the development of the dissemination materials. Facilitator-led small group discussions were used to finalise the content of the leaflet and how many topics would be included. Workshop participants also undertook filmed interviews and were recorded talking about the topics. This was so the recordings could be used in the film and incorporated into text for the leaflet. The young PLHIV reviewed the script for the film and discussed the film content and using a piece of art work. At the end of the workshop the young PLHIV had completed the filming and discussed how they would disseminate the leaflet and film to the wider HIV community. This workshop was evaluated by the workshop participants writing comments on sticky notes in response to a selection of questions. These included how they felt the weekend went, what could have been done better and whether they felt supported and listened to.

### Dissemination workshop 4

A final dissemination workshop in April 2019 was carried out involving three young PLHIV who had participated in the earlier workshops, (Group 1) and seven who had not participated before (Group 2). The aim was to confirm the most important concepts and results for communication through facilitator-led questions between the two groups of participants and review the proposed draft version of the leaflet to provide a final round of feedback and suggestions. The film maker and graphic designer then completed the film and leaflets.

## Results

**Fig. 1:**
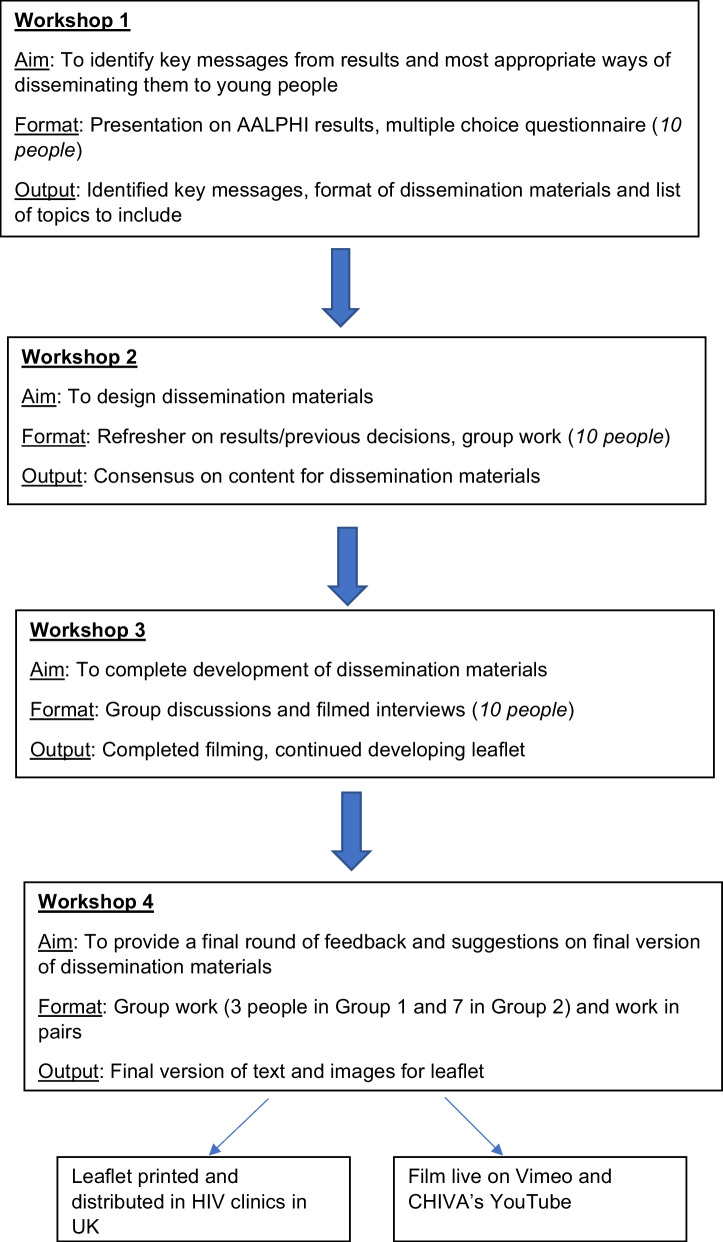
Flowchart of workshops

Ten young people participated in each dissemination workshop and were aged between 15 and 21 years, with 5 females and 5 males. They were all young PLHIV and on antiretroviral treatment. The same 10 PLHIV participated in the first three workshops and then three of them and seven new PLHIV participated in the final workshop so there was a total of fourteen people in the project overall. The structure of the workshops and their outputs is summarised in Fig. 1. Over four workshops the young people produced content for a leaflet and film. Each workshop built on the discussions and work from the previous one.

### Workshop 1:

By the end of the first workshop the young peoples’ knowledge of the AALPHI study results had improved as they all did better in the multiple choice questionnaire and they had written the key messages in a format and language that was understandable for young people. The participants decided that 3 leaflets on (1) medication and adherence; (2) mental health; anxiety/mood and how your brain is doing and (3) transition from paediatric to adult care, as well as a short film detailing the results, were the best ways to communicate the AALPHI results.

### Workshop 2

The young people were divided into pairs or threes and were asked to discuss the leaflet topics and what information they thought should be prioritised. The group were then brought back together in plenary and participants were invited to feedback briefly, the aim being to get a consensus view. Participants were encouraged to discuss the intended audiences for both the leaflets and the short film. They particularly focused on how to reach more isolated groups who are harder to reach and what might need to be done differently for them.

The group was then again split into 2 to 3 smaller groups and each group was asked to brainstorm some ideas on the design of the leaflet and film. They were asked to think about the needs of the key audiences (AALPHI research participants and young people affected by HIV or PLHIV) and then key words of what that might appeal to them, eg, bright, fun, informative. These key words were used to inform the development of a mood board.

The young PLHIV used a variety of magazines and other source materials for their mood board. The graphic designer and filmmaker discussed ideas generated for the content for the leaflets and the short film. The participants were then brought back as one group to discuss their ideas and the key information they wanted to include. The group shared their prioritised ideas. Facilitators sought to build consensus; including identifying any trends of similar lower priority ideas so that these were not lost. These ideas were then to be used by the graphic designer to develop the leaflets.

**Fig. 2:**
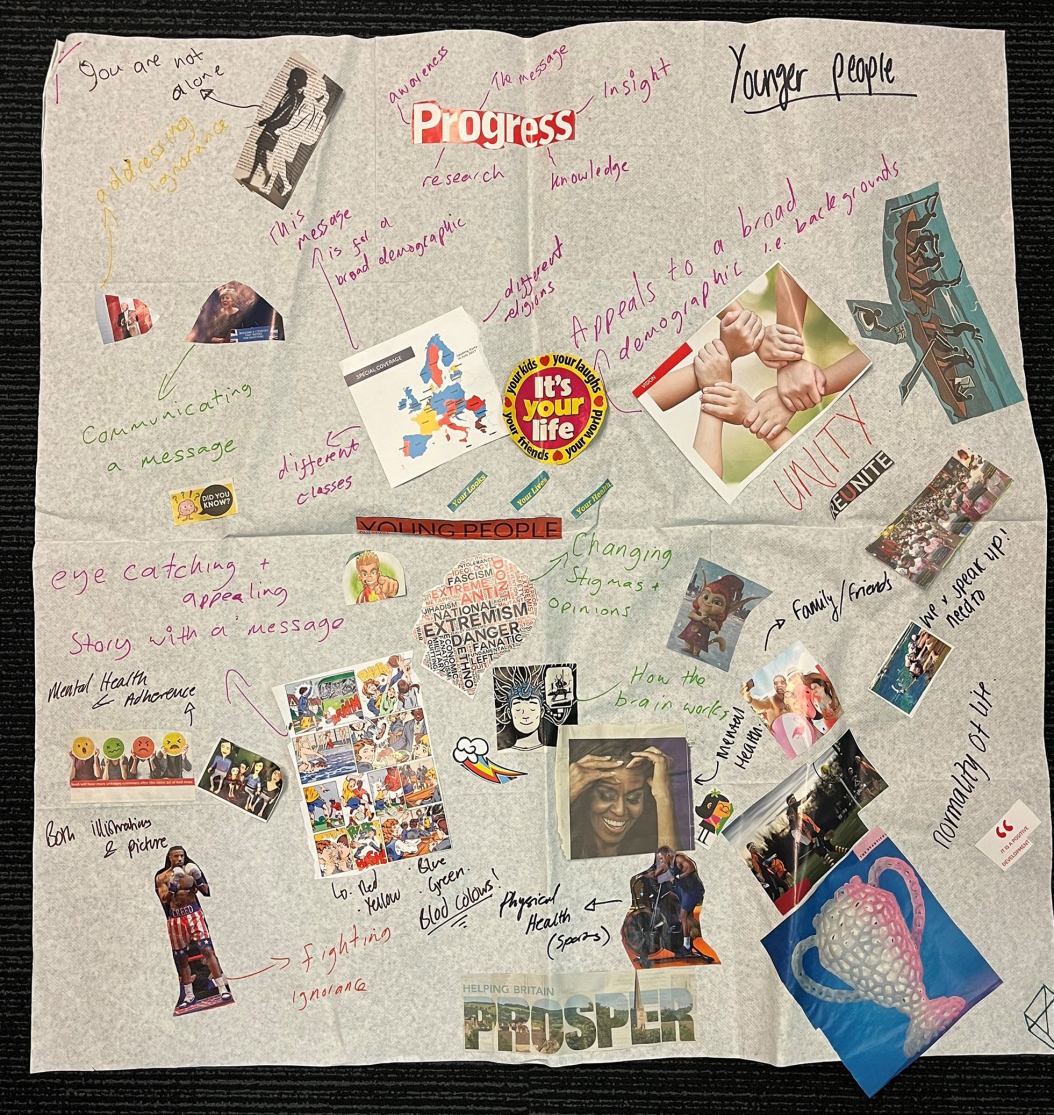
Mood Board 1 created by workshop participants

**Fig. 3:**
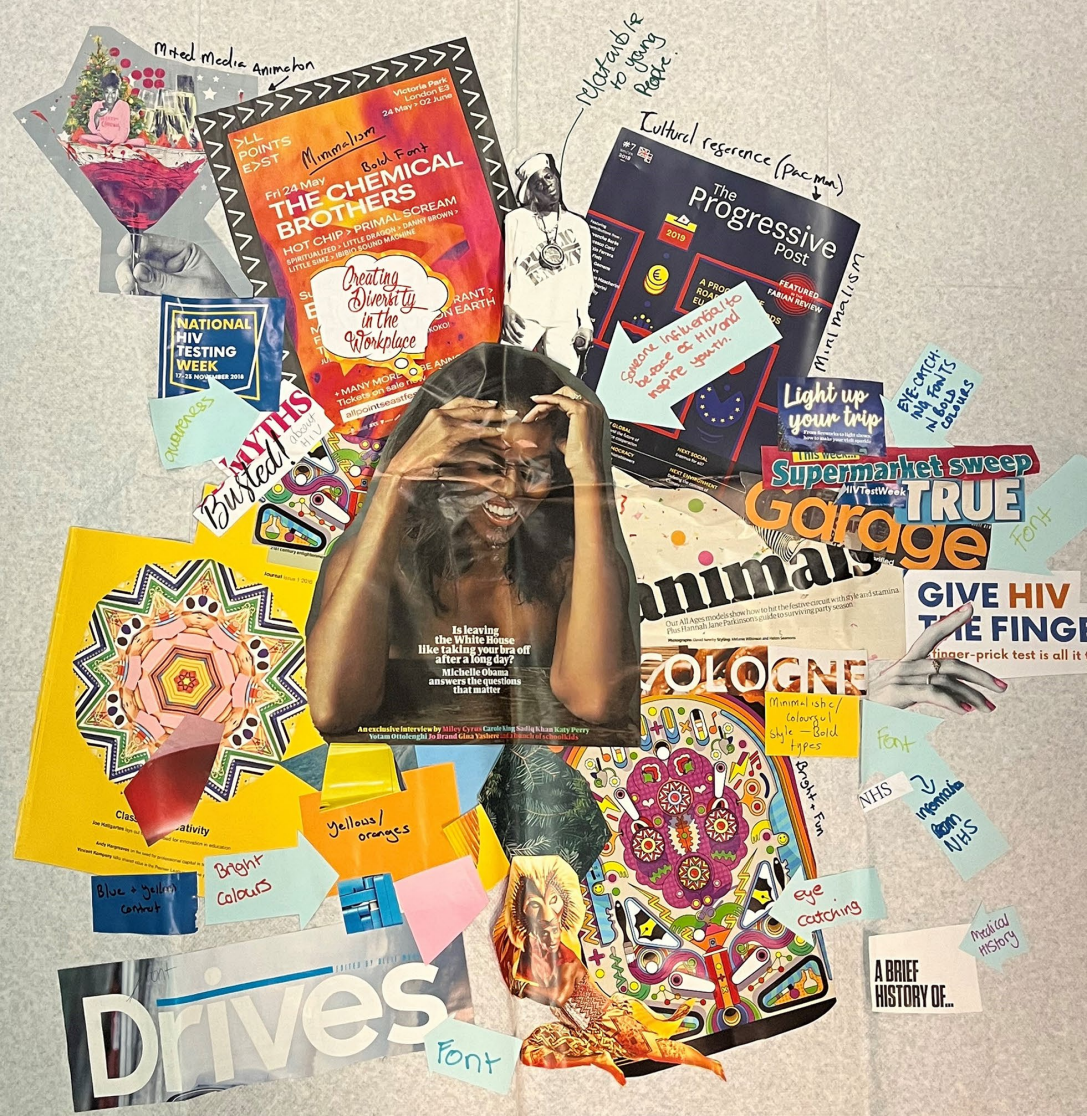
Mood Board 2 created by workshop participants

The graphic designer and film maker used the ‘mood boards’ (see Figs. 2 and 3), developed by the young PLHIV, in order to develop their requested style, colour and content for the leaflets and film. On the second day of Workshop 2 the young PLHIV developed the initial content of the leaflet and started to think about how the film would work with the film maker. They identified four main audiences to target for communication: AALPHI research participants, other young people living with HIV and/or young people affected by HIV, healthcare workers caring for young people living with HIV, and HIV treatment activist groups. They also discussed how there may be other audiences, but these would be informed via other routes, such as the annual CHIVA conference. The young people decided it would be better to make one set of leaflets for all age groups. By the end of Workshop 2 they had the initial content for the leaflets and some ideas about how they would do the film while disguising identities. This was important as most of the participants were not open about their HIV status.

### Workshop 3

In February 2019 Workshop 3 was held, the final residential weekend, to complete filming and continue developing the leaflets. The session again started with a refresher introductory briefing on the study and previous decisions on the project outputs. The young people were split into pairs or threes to talk about the AALPHI findings. The young people decided that the leaflet would include the 5 topics; medication and adherence; mental health (depression and anxiety); cognition (‘how your brain is doing’); sexual behaviour and sexual risk taking and transition from paediatric to adult care. This had changed from Workshop 1 where they had decided on 3 topics. For each leaflet topic they were given a task to help generate content but also build confidence to share their views with the graphic designer.

For the medication and adherence topic the participants were asked to give examples of their own experiences or instances such as when they have forgotten or chosen not to take their pills. They were also asked what has worked for them to remember and examples of what they have been told about why adherence is important. For the mental health topic a long piece of parcel tape was stuck on the wall with signs on either end saying “Positive mental health” and “Negative mental health”. There were multiple sheets of A3 paper, each with a printed header, and the group was asked to create a range of “heads” which signified what it is like to be a person living with HIV with positive mental health and negative mental health. Participants were asked to decorate their heads in a range of creative ways (e.g. drawing, writing, collage). From this stimulus, they considered the different services accessed by PLHIV which impacted on mental health, and the factors affecting PLHIV mental health.

For the transition topics they were asked to think about the best and worst possible experience of PLHIV at different ages from 5 to 15 years and what might need to change to ensure a smooth transition to adult care. The facilitators and the filmmaker then circulated between the groups, looking for visual examples to use.

In parallel to this work, participants were individually interviewed and recorded talking about these topics. One of the young people also helped with this and accompanied the film maker and other facilitators to ask questions. The young people also reviewed the script for the short film and the content for a rap song which the film maker had written and changed the words and the music for the rap song. Lastly, ideas for disseminating the leaflets and the short film were discussed by the participants as a group.

**Fig. 4:**
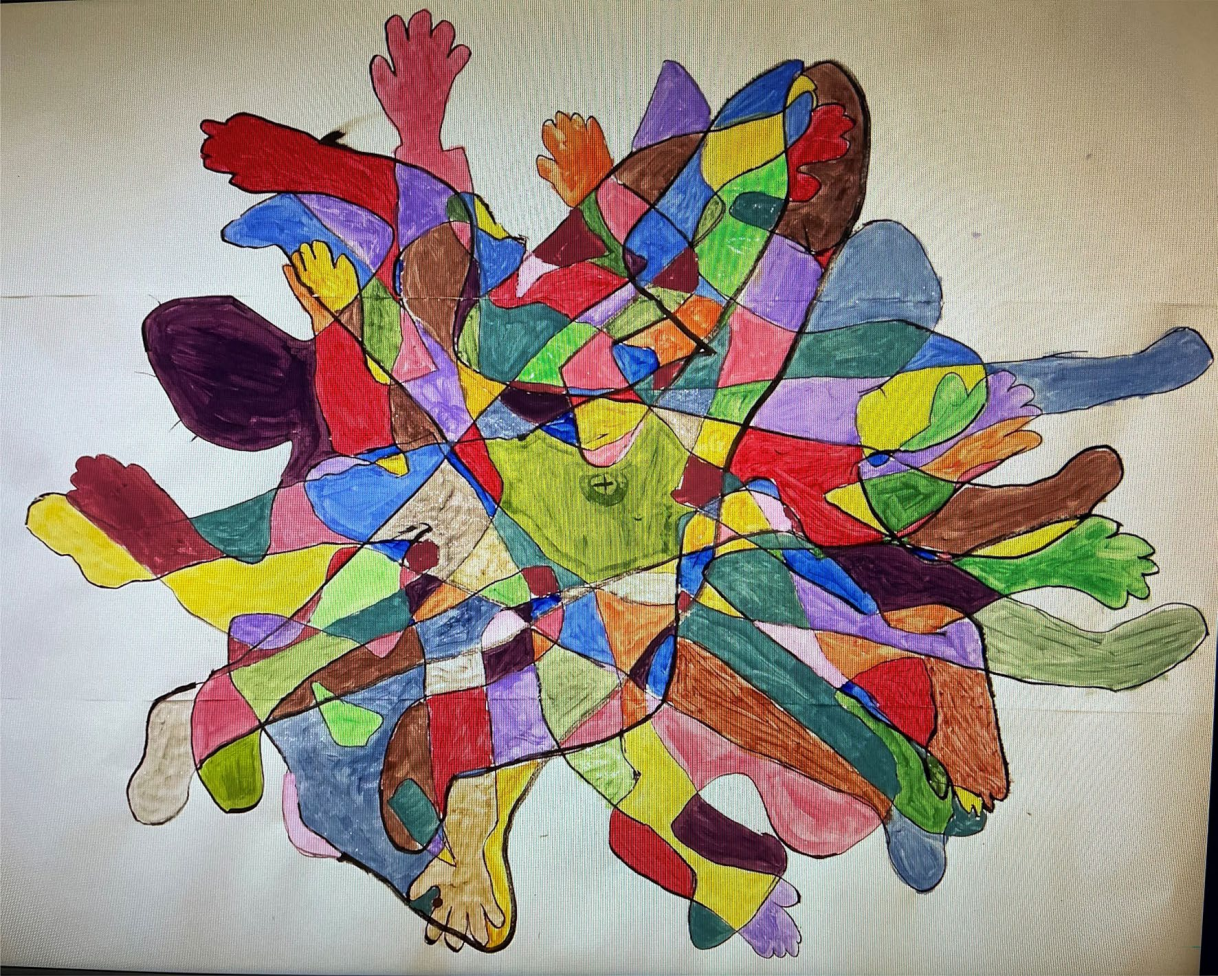
Painting by workshop participants to be used in the film

A piece of art work was created to use in the film. The tallest participant laid down on a large sheet of paper, positioned with their navel in the middle of the sheet, and their body outline traced around. All participants followed suit but at different angles, until everyone had been traced, and then poster paints were used to ‘fill in’ the bodies (see Fig. 4).

The interesting examples from the group work and the quotes from the recordings of their voices talking about their experiences of living with HIV were incorporated into the script for the film and the text of the leaflet. They addressed the topics from the AALPHI findings, such as how they find taking medication and how they manage feeling depressed or anxious. Most of the young people wanted to disguise their faces and voices, therefore they were filmed as silhouettes behind a large sheet talking through the script. By the end of Workshop 3 they had completed all the filming. The workshop finished with some discussion around how to disseminate the outputs, e.g, getting the film and leaflets into spaces occupied by the wider HIV community.

The feedback from participants included how they felt the weekend went, what could have been done better and whether they felt supported, listened to and involved in the decision-making processes. There were lots of positive comments about the weekend, such as ‘productive’, ‘informative and important’ and ‘well organised’. Participants reported that they had received great support from staff, felt listened to and had gained confidence. A couple of young people felt they needed to be listened to a bit more when developing the film, and some felt that the venue could have been better and that more time was needed, (see Table 1).

**Table 1:** Feedback gathered from the 10 workshop participants

How do you feel this weekend went?	How could we do this better?	Did you feel supported and listened to and involved in discussions?	What did you think about the venue?	Was there enough time?
Very productive, learnt lots and met new people	More game time	Great support from staff. Decisions were made collectively and everyone could voice their opinions	Too formal and a bit intimidating	A bit more time would have been better
Informative and important	More group activities together	It was good, gained confidence and could express your views and what you believe	Venue was great, little dry but a lovely working environment	Needed 3 days rather than 2
Exhausting but very fun ad worth it!	Make sure no one is missing out on activities	Listened to very well throughout	Would like better ventilation	It was too short, we had to cover things very quickly
Well organised	Need to be heard a little better film wise	Needed to be listened to a little better film wise	Fancy venue	Perfect length, the 2 days away were great
Highlights – rounding up the study and the games we played		We felt supported	The venue was great, the rooms were a bit cold	Loved it, would not change it!
Highlight—working with the graphic designer				Good amount of time as there was lots to do

Workshops 2 and 3 were held as residential weekends due to the large volume of work that needed to be done, therefore apart from two participants who lived in London, the rest of the group spent two nights and two days together over each workshop. This was therefore quite an intense environment for the young PLHIV to be in, however, the facilitators stayed with them overnight so they were supported throughout the weekends. This provided pastoral care and was also important as some young people talked to the facilitators outside of the workshops about what was not going as well, such as they felt the need to be heard more when making the film, (see Table 1). For some of the participants who were not open about their HIV status, this was a unique experience as it was the first time they were in a supportive environment with other young PLHIV.

It was observed that the participants had created their own youth group. Eight of the ten young PLHIV knew each other before through the YTB UK and CHIVA networks but two of the AALPHI participants did not know anyone. The group developed as the workshops progressed and reflected Tuckman’s stages of group development [13]. In the beginning, known as the ‘forming stage’ the individuals seemed unsure of how they fitted in and were polite and tentatively joining in. As time went on there were more differences in points of view. This stage, called ‘storming’ normally has power struggles, clashes and demonstrates a lack of progress but this was not seen. However, there was a definite ‘norming’ stage that followed where the young PHLIV were comfortable with the relationships, there was shared problem solving and task milestones were achieved, such as making decisions about the leaflet and film content. By Workshop 3 the group was working together really well and demonstrated independence when they changed the words and music for the rap song and were better able to organise themselves. Tuckman calls this the ‘performing’ stage. The final ‘Adjourning’ stage is when the group members leave and there are visible ‘signs of grief’. This was observed after Workshop 3 when the same group were not going to continue to work together and they expressed that they were sad to leave each other.

### Workshop 4

In April 2019, a half-day workshop was held at the CHIVA offices in Bristol, UK, to finalise the text and graphic images for the leaflet. Participants in the workshop included 3 who had been previously involved in developing dissemination materials (Group 1) and a new group of 7 young people who had not previously been involved (Group 2). As an initial exercise to consider audience needs and explain the AALPHI findings, Group 1 drafted a list of questions to ask to Group 2 about AALPHI findings, and Group 2 were given the results from AALPHI and created some facts as well as some false information. Group 1 asked their questions to Groups 2, and then Group 2 (with support from facilitators) answered all the questions, but were allowed to give false information about the study. Group 1 was challenged with guessing which facts were true and which were fake, with points awarded for correct answers. Afterwards, the facilitators corrected any false information and gave an overview of the accurate AALPHI findings.

The facilitators then explained decisions made to date on the audience for the leaflet, the mood boards and the format. In two groups they discussed the features of good and bad leaflets, and why. Ideas were recorded on a flipchart, as a check list to use to review the AALPHI leaflets later. Everyone fed back in a plenary on key aspects which contribute to good leaflet design (e.g. simple language, pictures), and this was recorded on a flipchart. It was decided that the leaflet should have 8 sides or panels; 5 for the key topics, a cover panel and another giving some background on the AALPHI study and the back panel with where to find more information on other HIV adolescent studies.

Finally participants were divided into pairs and each pair was given one of the AALPHI themes. The pairs had draft sections developed by a graphic designer based on previous workshop results. The pairs reviewed and decided what text they wanted on each panel, using information from the script that had been written in Workshop 3 and the quotes from the recordings. Together they cut out the text or rewrote it along with drawing their own ideas for illustrations for each. They then came back as a group and presented their leaflet or panels and each was discussed against the decisions that were agreed earlier in the earlier dissemination workshops on audience, mood boards and format.

**Fig. 5:**
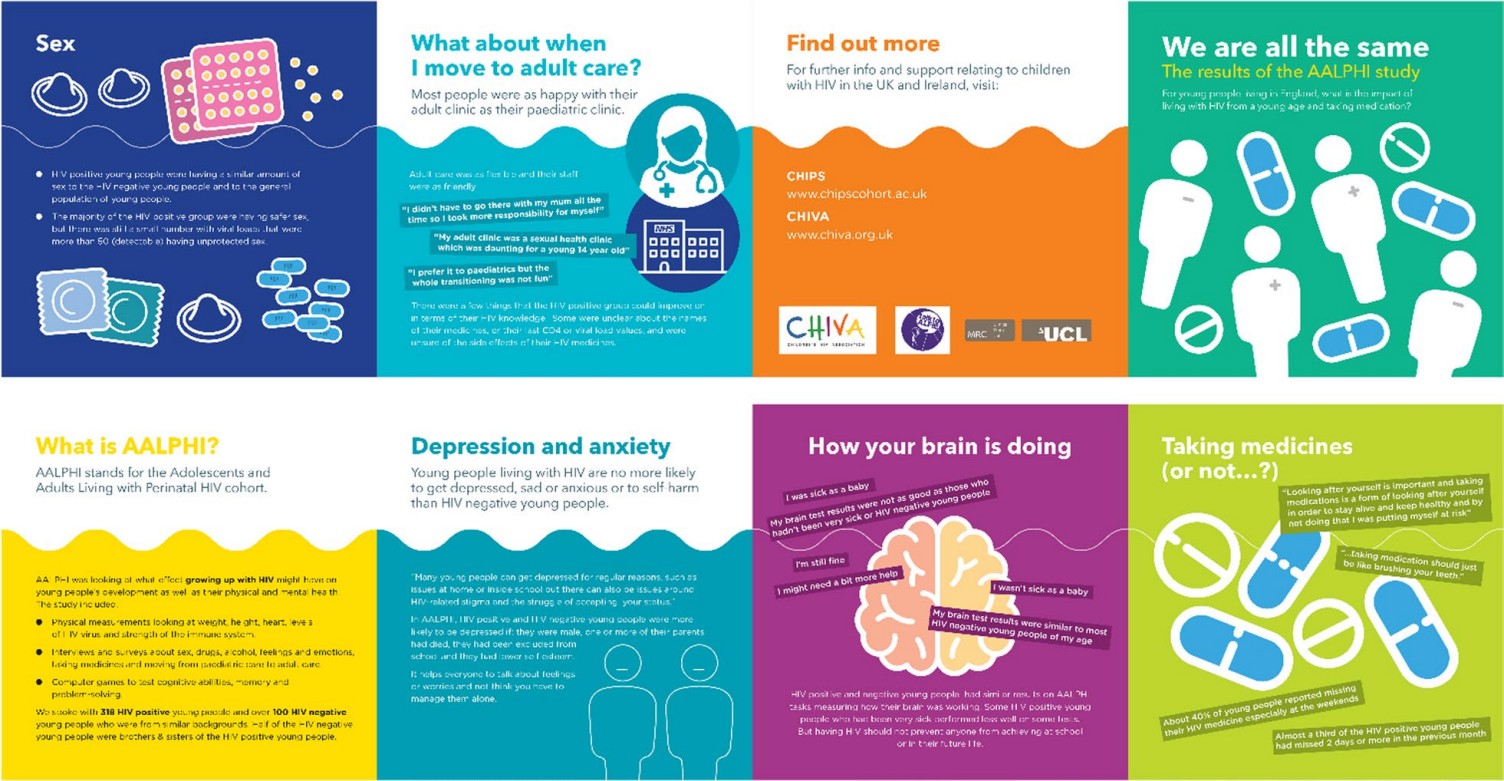
Leaflet developed by workshop participants

By the end of Workshop 4, a final version of the text and images for the leaflet had been produced, which the film maker and graphic designer used to then develop the leaflet and film. Over the next few months, all the young people who had participated in all four workshops and had agreed to review the film and leaflet were sent drafts to comment on. By the end of November 2019 the leaflet was printed (see Fig. 5) and the film was live on Vimeo.

### Dissemination materials

The leaflet, (see Fig. 5), was widely distributed in HIV clinics across the UK. It was originally planned for the young people from the project to attend some HIV clinics in London to disseminate the leaflet and talk to young people about it. However, due to the SARS-CoV-2 pandemic this was not possible. Instead, the leaflet was printed and copies were posted to clinics, and the leaflet and film were made available on CHIVA’s social media and website, [14].

One of the young people presented the project at the CHIVA conference in London in April 2019 and another young person presented it at the Heath Care and Research Conference in Wales in September 2019. The project was also discussed at an HIV Young Person’s Network (HYPNet), meeting. HYPNet is a multidisciplinary group of health professionals and voluntary sector representatives working with young PLHIV aged 13–24 years, largely in the south-east of England.

## Discussion

This project demonstrates the advantages of involvement of young people as stakeholders in the development of dissemination materials for study results. It ensured that the dissemination materials for the AALPHI study were developed by young people, for young people. Previous work has shown that PPI and co-production improves the quality of dissemination [15, 16]. Over the course of four workshops, PLHIV, including some AALPHI participants, worked together and chose how to frame the key messages from the AALPHI findings and how to disseminate them. The main outputs of the project were a leaflet and a short film, made in collaboration with a graphic designer and film maker. The project was later presented by two young people at two conferences.

The participants in this project (unintendedly) created their own youth group. This was not expected but over time it had its own group dynamic and power relations. It was observed there is something powerful about a residential, especially amongst participants who are often not open about their HIV status as it gave them the opportunity to be in a supportive space with other positive peers. Others planning dissemination may wish to plan for the psychosocial support needs and the long-term nature of group dynamics and being in intensive residential spaces.

Taking part in workshops, especially for those who took part in multiple workshops, meant the young PLHIV had much greater contact with researchers and developed knowledge of the AALPHI study findings and of research methods/processes. They will have had a very in-depth understanding that their own health (at least as measured in AALPHI) was generally comparable to HIV affected peers in close relationship with an HIV positive individual, [4, 5].

There was a power dynamic between the participants, the facilitators and the film maker and graphic designer. The biggest conflict at the end was the young PLHIV not agreeing with the creative’s approach and the facilitators trying to balance getting a project done (to time, budget, etc.) and authentically protecting the young PLHIVs’ views and opinions. In a small group one or two young people who dislike the creatives’ ideas can have a big impact. The challenge of a power imbalance has been cited in other research, such as youth participation in mental health research, where researchers were concerned about the impact these dynamics can potentially have on a young person [17]. Therefore there can be wider issues for facilitators of PPI processes and setting clear boundaries is really important. Creatives may benefit from training on the process of co-production and how to manage a group’s dynamics.

The environment that participatory activities are held in is important for young people. In a recently published report on quality standards for adolescent participation in clinical research decisions-making, the environment is acknowledged as an important factor in the delivery and management of any participatory activity. The report’s authors recommend the following: “Staff create a welcoming and accessible environment for adolescents. Everyone is made aware of expectations of the way they should behave in this environment, (e.g. Codes of Conduct or Ways of Working)’, [1]. The “Meaningful Involvement of People with HIV/ AIDS” [18] explores potential barriers to the meaningful involvement of people in an organisation and highlights the importance of the physical space. This includes if the staff are accessible to the clients or community and if there is comfortable seating, [18].

All 10 young people wanted to make a film and disseminate it on social media and it is well known this is how many young people like to engage with the world. However, little research has been done to explore whether social media is an effective platform to engage young people in the results of trials and studies. One study that looked at young people’s experiences and perceptions of YouTube health content, confirmed YouTube as a successful platform to communicate health messages to young people. In that study focus groups were conducted with 85 young people (13–18 years) and they found that YouTube health content was one of the many sources of health information used by young people and was most frequently seen during young people’s routine viewing [19].

The film was released on Vimeo and recent feedback from a young person who participated in the project was that this was not the best social media platform to use to disseminate the film. Adolescents do not commonly use Vimeo and it would have been better to use YouTube which is widely accessed by this age group. However, the MRC CTU at UCL does not have patient-facing social media accounts. Thus, the video was posted on CHIVA’s YouTube platform, which is mainly used by young PLHIV and people affected by HIV. This highlights the importance of social media as a platform to get information out to young people as this is how adolescents regularly communicate with each other and explore the world.

However, further exploration into how to successfully use social media to communicate research findings to young people is required. Focus groups with young people to discuss which social media platforms are most accessible to young people and the best format of the information to deliver messages, such as videos versus infographics, could be helpful.

## Limitations

The majority of the feedback from the young people at the end of the project was positive, (see Table 1), however there were some challenges.

Feedback from participants also included that the office space was intimidating for some, making the workshops feel like a work meeting, and participants would have preferred a more relaxed venue. Some participants also felt they needed to have more control while working with professional film makers, (see Table 1). The venue was chosen to save on costs but a more appropriate venue would be recommended if funding was not an issue. It was challenging making a film over two weekends and disguising identities however these barriers were overcome so that everyone could be involved and feature in the film.

Finally, there was limited ownership of the final dissemination products for the seven young people that did not participant in the fourth workshop and therefore were not part of the final decisions made about the leaflet and film.

## Conclusion

PPI is critical in the dissemination of research findings that are relevant to young people. This project has shown it is feasible even with groups with additional considerations, such as young people affected by a chronic condition, and often subject to societal stigma. The development of the key messages from AALPHI into materials that reflected the priorities expressed by this sample of the target patient group could not have been done without them. The intention is that this will make them be better advocates for their own health care, as other evidence suggests [20]. This project has shown how researchers can use imaginative ways of involving people/patients in areas such as HIV and young people and the creative materials that can be produced when engaging young people in the dissemination process.

## Data Availability

Not applicable.

## Abbreviations

AALPHI: Adolescents and adults living with perinatal HIV
PLHIV: Young people living with perinatal HIV
YTB UK: Youth Trials Board UK
CHIVA: Children’s HIV Association
PPI: Patient and Public Involvement
